# Detection of unrecognized pregnancy prior to a fluoroscopy‐guided interventional procedure: A case report

**DOI:** 10.1002/ccr3.2437

**Published:** 2019-10-06

**Authors:** Semih Gungor, Ersu Celebi

**Affiliations:** ^1^ Department of Anesthesiology, Critical Care and Pain Management Hospital for Special Surgery New York NY USA; ^2^ Department of Anesthesiology Weill Cornell Medicine New York NY USA; ^3^ Department of Anesthesiology Faculty of Medicine Hacettepe University Ankara Turkey

**Keywords:** fluoroscopy, hCG, intervention, pain, pregnancy, urine test, x‐ray

## Abstract

The aim of this case report is to increase the awareness about patient and fetus safety through preprocedure assessment and screening of unrecognized pregnancy for fluoroscopy‐guided procedures.

## Graphical abstract

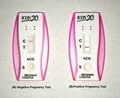

## 1 INTRODUCTION

Fluoroscopy‐guided interventional pain management procedures are performed commonly for the diagnosis and treatment of various pain conditions. It is well known that radiation exposure during pregnancy can cause detrimental effects on fetus.^1^, ^2^, ^3^, ^4^ However, not all pregnant women who are scheduled to undergo fluoroscopy‐guided interventions are aware of their pregnancy.^5^ Particularly, the subtlety of early signs and symptoms of pregnancy may result in an unrecognized pregnancy at the time of presentation for a fluoroscopy‐guided procedure. Embryo is most susceptible to radiation exposure during organogenesis. Some of the risks of in utero radiation exposure are prenatal death, growth retardation, organ malformation such as small head/brain size, mental retardation, intellectual (IQ) disability, neocortical ectopias, callosal agenesis, and childhood tumors.^2^, ^3^, ^4^ There are general practice guidelines published by American College of Radiology (ACR) for imaging pregnant or potentially pregnant patients.^4^ However, there has been no universal consensus in regard to screening or performing routine pregnancy testing prior to performing interventional pain management procedures. At our institution, a policy for preoperative urine pregnancy testing for surgical patients requiring intravenous anesthesia and surgery was first implemented in 2004.^6^ Subsequently, a similar policy was also implemented in 2011 for fluoroscopy‐guided interventional pain management procedures which do not require intravenous anesthetics. We describe a case report of an unrecognized pregnancy diagnosed with the routine preprocedure rapid urine pregnancy test, which resulted in cancelation of the fluoroscopy‐guided procedure. The aim of this case report is to increase the awareness about patient and fetus safety through preprocedure assessment and screening for unrecognized pregnancy for fluoroscopy‐guided procedures.

## 2 CASE REPORT

A 31‐year‐old female patient, who was a high‐level medical professional, was scheduled for lumbar epidural steroid injection in our institution. The authors have obtained written consent to publish this case report from the patient. Institutional Review Board approval was obtained for this case report on 23 October 2018 (IRB# 2018‐1980). The patient was complaining of severe left‐sided lower extremity radicular pain for over 3‐month duration. In this particular case, the patient had failed conservative therapy with activity modification, home exercises, medication management, and physical therapy for at least 3‐month duration. The patient was evaluated by a spine surgeon, and epidural steroid injection was recommended prior to considering an elective spinal surgery. Fluoroscopy‐guided interventional procedures are used for the treatment of painful conditions after the conservative therapy fails. Therefore, to improve the pain and the activities of daily living, as well as to avoid any unnecessary spinal surgery, fluoroscopy‐guided epidural steroid injection was scheduled.

**Figure 1:**
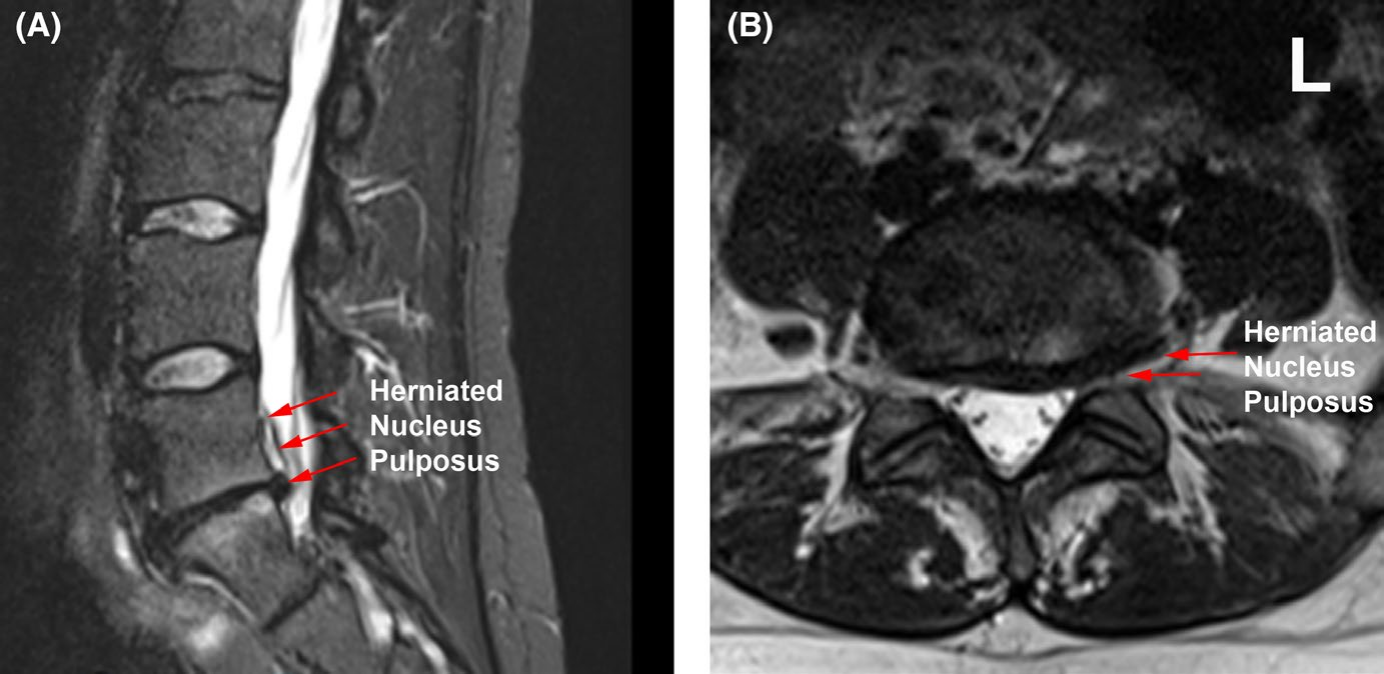
Lumbar spine MRI without contrast showing a disk herniation at L5‐S1 level. A, Sagittal MRI: T2‐weighted STIR (Short‐TI Inversion Recovery), B, axial MRI: T2‐weighted

**Figure 2:**
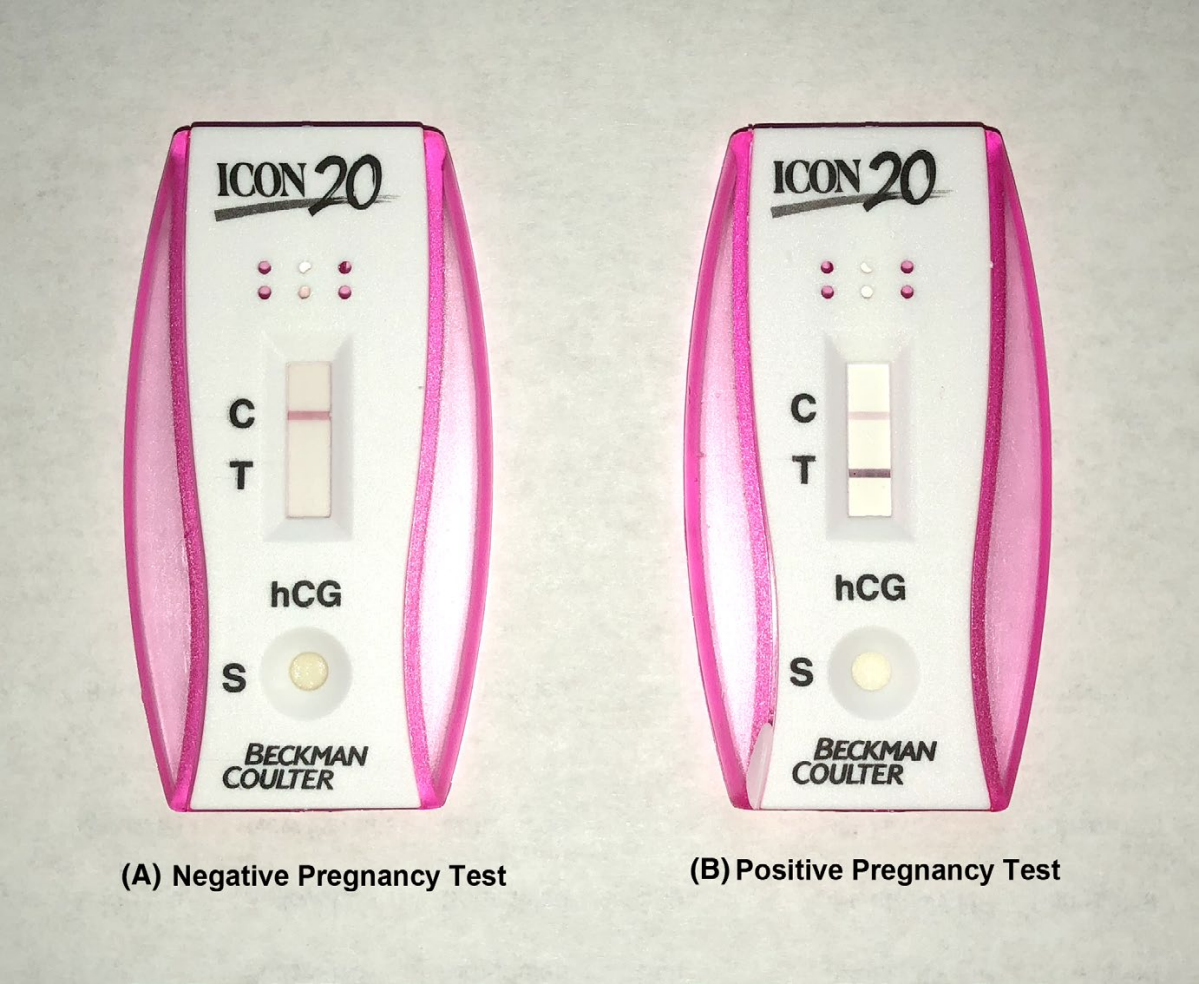
Sample of rapid point‐of‐care urine pregnancy test (A, negative, B, positive)

**Figure 3:**
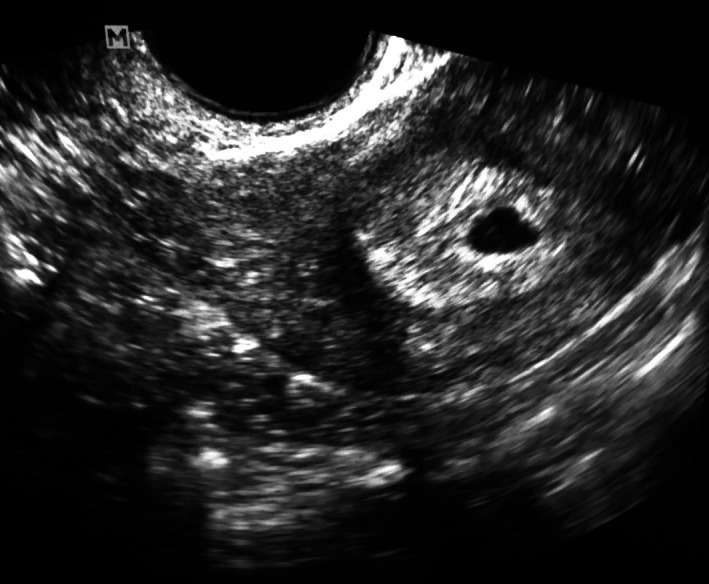
Pelvic ultrasound examination of the patient showing a fetus of 6 wk of pregnancy

The patient's neurological examination was consistent with left L5 and S1 radiculopathy. MRI of the lumbar spine showed large disk herniation at left L5‐S1 level (Figure 1). As per the policy, the patient was asked the routine preprocedure question of possibility of being pregnant at that time, and the answer from the patient was “no.” Upon arrival in the preprocedure holding area, the patient underwent routine preparation including rapid qualitative point‐of‐care urine pregnancy test. Urine pregnancy test showed positive result for an unrecognized pregnancy (Figure 2). The patient was informed about her pregnancy and possible effects of radiation exposure on fetus. The procedure was canceled. The patient was referred to her obstetrician. Upon follow‐up with her obstetrician, 6 weeks of pregnancy was confirmed by serum pregnancy test and by pelvic ultrasound examination (Figure 3). The patient returned for follow‐up with similar pain and symptoms 3 months after this episode. The patient reported us that she had had a spontaneous miscarriage around 18 weeks of pregnancy for unknown reasons. After following through the same preprocedure protocol, including a negative urine pregnancy test result this time, the patient underwent successful left L5 and S1 transforaminal epidural steroid injection under fluoroscopic guidance with over 90% improvement of pain and symptoms during further follow‐ups.

## 3 MATERIALS AND METHOD

Due to medico‐legal importance of this topic, our hospital has implemented a policy in regard to possible diagnosis of unknown pregnancy prior to fluoroscopy‐guided pain management procedures. All female patients of childbearing age who are scheduled for fluoroscopy‐guided pain management procedures are asked the question about a possible or absolute pregnancy 1‐3 days prior to the planned procedure. If the answer is “yes” for absolute pregnancy, the procedure is canceled, and the medical staff is informed. If the answer is “no” after the preoperative telephone conversation, the patients are allowed to come for the procedure on the scheduled day. Upon arrival to preprocedure area on the date of scheduled procedure, all female patients of childbearing age are again asked for possible or absolute pregnancy. If the answer is “yes” for absolute pregnancy, the procedure is canceled, and the medical staff is informed. If the answer is “no,” as outlined in the hospital policy, the eligible patients will have a point‐of‐care rapid urine pregnancy testing.

At our institution, as directed by the policy, a urine specimen is obtained from all females of childbearing age upon arrival in the preprocedure holding area. Preprocedure medications are held until the urine pregnancy test results are available. Exclusion criteria for urine pregnancy testing are menopause (defined as the age between initial reported menses, and 1 year after last reported menses), history of hysterectomy or bilateral salpingo‐oophorectomy, and patient refusal. The reported uses of any contraceptive medication/device or history of tubal ligation are not considered as the criteria for exclusion from testing. No one of the procedure team is authorized to waive a pregnancy test. The patient is the only individual who may elect to waive a pregnancy test after being advised of the associated risks. However, this decision requires documentation in the chart. In the event of a positive urine pregnancy test, the attending physician notifies the patient, followed by a cancelation of elective procedure. The patient is then referred to an obstetrician.^6^

The rapid qualitative point‐of‐care urine pregnancy test used in our institution was a simple immunoassay for the qualitative detection of hCG (human chorionic gonodotropin) in the urine sample (ICON^®^20 hCG, Beckman Coulter). The test employs solid‐phase chromatographic immunoassay technology with mouse anti‐hCG monoclonal antibodies to selectively detect elevated levels of hCG. After exposure to the test reagents, specimens produce a color change on the assay membrane if they contain hCG (Figure 2).

The test is performed by introducing drops of urine sample on the indicated site of the kit and the result is readable within 3‐5 minutes. These tests usually contain a strip impregnated with anti‐hCG globulin and a color indicator. If the urine sample was appropriately tested, there will be a single‐line color change appearing as the control, indicating a valid test. If a double‐line color change appears (one for control and one for positive hCG level), the test is then interpreted as positive for pregnancy (Figure 2).

## 4 DISCUSSION

Epidural steroid injection is not considered as a first‐line therapy for lumbar radiculopathy and should be considered after the conservative therapies fail. The risks of epidural steroids injection are radiation exposure, procedural and/or medication‐related side effects or complications.

It is well known that radiation exposure during pregnancy can cause detrimental effects on fetus.^1^, ^2^, ^3^, ^4^ A number of pregnancies could be unknown at the time of presentation for a fluoroscopy‐guided procedure.^5^ Pregnancy cannot be confirmed or ruled out by patient history alone.^5^ Particularly in early pregnancy, the subtlety of early signs and symptoms of pregnancy, a history of irregular menses, the use of contraceptives, and misconceptions regarding pregnancy may result in an unrecognized pregnancy by the patient. Embryo is most susceptible to radiation exposure during organogenesis. Consequences may vary with the stage of pregnancy and total radiation dose absorbed by the fetus. In utero radiation exposure could result in prenatal death, growth retardation, organ malformation such as small head/brain size, mental retardation, intellectual (IQ) disability, neocortical ectopias, callosal agenesis, and childhood tumors.^2^, ^3^, ^4^ As per the Center for Disease Prevention Center (CDC) website, the prevalence of intellectual disability (IQ < 70) is 40% after an exposure of 1 Gy from 8th to 15th week, and the prevalence of intellectual disability (IQ < 70) is 15% after an exposure of 1 Gy from 16th to 25th week.^7^

Therefore, recognition of pregnancy before fluoroscopy‐guided procedures is critical in preventing fetal radiation exposure, and thus to avoid potential medical, psychological, and legal consequences.

As per the ACR practice guidelines, for procedures that are expected to involve an unpredictable duration of fluoroscopy especially for the body parts in close proximity to pelvis, such as lumbar‐sacral spinal interventions, it is recommended that all female patients of childbearing age should be screened by questioning and/or a pregnancy test should be obtained within 72 hours prior to commencement of the procedure unless medical exigencies prevent it.^4^

As directed by the policy at our institution, urine pregnancy tests are routinely performed prior to fluoroscopy‐guided interventional pain procedures in women of childbearing age, unless a history of menopause, prior hysterectomy or bilateral salpingo‐oophorectomy are reported or documented.

Pregnancy tests are based on the measurement of elevated levels of hCG, which is a hormone produced by placenta as early as 7‐10 days after fertilization. The detection of hCG in urine is an easy first method of diagnosing pregnancy. Rapid qualitative point‐of‐care urine pregnancy detection kits are readily available in the market and can be used on‐site prior to the scheduled procedure. Sensitivity and specificity of urine hCG are found to be 99% and 92.2%, respectively. Although the test could result false positive or false negative, such outcomes are infrequent and can easily be corrected by serum tests.^8^

A retrospective chart review by Kahn et al^6^ found 5 positive test results out of 2588 women, which is an incidence of 0.2%, after the implementation of urine hCG testing for all women of childbearing age on the day of surgery in our institution.

Although American Society of Anesthesiologists recommends pregnancy testing to be offered to female patients of childbearing age before anesthesia,^9^ and ACR practice guidelines recommend that all female patients of childbearing age should be screened by questioning and/or a pregnancy test should be obtained within 72 hours prior to the procedure,^4^ we have not been able to find any published guideline or consensus statement regarding pregnancy testing specifically for women prior to fluoroscopy‐guided interventional pain management procedures.

In our case, the patient had a spontaneous miscarriage approximately around 18 weeks of gestation. This incident was spontaneous for unknown reasons but without any exposure to radiation as the procedure had been canceled after finding out that the patient was 6‐week pregnant. However, the psychological, medical, and legal consequences might have been different, had the patient been exposed to ionizing radiation without being diagnosed as pregnant at 6 weeks of gestation with a routine preprocedure rapid on‐site urine pregnancy test as directed by our hospital policy for pain management procedures.

Severe lumbosacral radiculopathy refractory to conservative therapy may be encountered during pregnancy. If the patient had had the continuation of pregnancy with progression of the radicular pain and symptoms, epidural steroid injection might have been considered in this particular patient in the later stages of pregnancy if indicated. As a safe and a real‐time imaging modality, ultrasound guidance may be considered during the performance of caudal epidural injections in feasible cases, specifically if the pathology is at the lower lumbar levels such as L5‐S1 level similar to pathology in this particular patient. The patient's obstetrician should be consulted and included in decision‐making process. While steroids can be administered to women in pregnancy since it may help fetal lung maturity, they should only be administered for short periods of time, since long‐term use poses high risk of negative fetal effects. Steroid preparations with normal doses are usually accepted as safe during the second and third trimester of pregnancy.

## 5 CONCLUSION

Radiation exposure during an unrecognized pregnancy may have medical, psychological, and legal consequences. Therefore, we recommend routine rapid qualitative point‐of‐care urine pregnancy test prior to fluoroscopy‐guided interventional procedures in all women of childbearing age, unless a history of menopause, prior hysterectomy or bilateral salpingo‐oophorectomy are reported or documented. Recognizing a pregnancy allows a woman and her physician to make an informed decision before proceeding with an elective procedure.

## CONFLICT OF INTEREST

None declared.

## AUTHOR CONTRIBUTIONS

SG and EC: contributed to performance of the case, chart review, manuscript preparation, and final approval of the manuscript.

## References

[1] Shaw P , Duncan A , Vouyouka A , Ozsvath K . Radiation exposure and pregnancy. J Vasc Surg. 2011;53(1):28‐34. 20869193 10.1016/j.jvs.2010.05.140

[2] Yang B , Ren BX , Tang FR . Prenatal irradiation–induced brain neuropathology and cognitive impairment. Brain Develop. 2017;39(1):10‐22. 10.1016/j.braindev.2016.07.008 27527732

[3] Sreetharan S , Thome C , Tharmalingam S , et al. Ionizing radiation exposure during pregnancy: effects on postnatal development and life. Radiat Res. 2017;187(6):647‐658. 28418814 10.1667/RR14657.1

[4] American College of Radiology . ACR Practice Guideline for Imaging Pregnant or Potentially Pregnant Adolescents and Women with Ionizing Radiation. Reston, VA: ACR; 2008.

[5] Ramoska EA , Sacchetti AD , Nepp M . Reliability of patient history in determining the possibility of pregnancy. Ann Emerg Med. 1989;18(1):48‐50. 2462800 10.1016/s0196-0644(89)80310-5

[6] Kahn RL , Stanton MA , Tong‐Ngork S , Liguori GA , Edmonds CR , Levine DS . One‐year experience with day‐of‐surgery pregnancy testing before elective orthopedic procedures. Anest Analg. 2008;106(4):1127‐1131. 10.1213/ane.0b013e31816788df 18349183

[7] Radiation and pregnancy: a fact sheet for clinicians . CDC: centers for disease control and prevention. http://www.cdc.gov/nceh/radiation/emergencies/prenatalphysician.htm. Accessed July 22, 2018.

[8] Montagnana M , Trenti T , Aloe R , Cervellin G , Lippi G . Human chorionic gonadotropin in pregnancy diagnostics. Clin Chim Acta. 2011;412(17‐18):1515‐1520. 21635878 10.1016/j.cca.2011.05.025

[9] http://www.asahq.org/quality-and-practice-management/standards-guidelines-and-related-resources/pregnancy-testing-prior-to-anesthesia-and-surgery. Accessed July 22, 2018.

